# The Importance of Social Cognition in Improving Functional Outcomes in Schizophrenia

**DOI:** 10.3389/fpsyt.2018.00157

**Published:** 2018-04-24

**Authors:** Afzal Javed, Asha Charles

**Affiliations:** ^1^Jepson House, Coventry and Warwickshire Partnership NHS Trust, Nuneaton, United Kingdom; ^2^Caludon Centre, Coventry and Warwickshire Partnership NHS Trust, Coventry, United Kingdom

**Keywords:** cognition, functional outcome, neurocognition, psychosocial intervention, recovery, schizophrenia, social cognition, social functioning

## Abstract

Social cognition has become recognized as an important driver of functional outcomes and overall recovery in patients with schizophrenia, mediating the relationship between neurocognition and social functioning. Since antipsychotic therapy targeting remission of clinical symptoms has been shown to have a limited impact on social cognition, there has been an increasing drive to develop therapeutic strategies to specifically improve social cognition in schizophrenia. We sought to review current evidence relating to social cognition in schizophrenia and its clinical implications, including interventions designed to target the core domains of social cognition (emotion processing, theory of mind, attributional bias, and social perception) as a means of improving functional outcomes and thereby increasing the likelihood of recovery. Relevant articles were identified by conducting a literature search in PubMed using the search terms “schizophrenia” AND “cognition” AND “social functioning,” limited to Title/Abstract, over a time period of the past 10 years. Current evidence demonstrates that schizophrenia is associated with impairments in all four core domains of social cognition, during the pre-first-episode, first-episode, early, and chronic phases of the disease, and that such impairments are important determinants of functional outcome. Interventions targeting the four core domains of social cognition comprise psychosocial approaches (social cognition training programs) and pharmacological therapies. Social cognition training programs targeting multiple and specific core domains of social cognition have shown promise in improving social cognition skills, which, in some cases, has translated into improvements in functional outcomes. Use of some psychosocial interventions has additionally resulted in improvements in clinical symptoms and/or quality of life. Pharmacological therapies, including oxytocin and certain antipsychotics, have yielded more mixed results, due in part to the confounding impact of factors including variation in receptor genetics, bioavailability, pharmacokinetics, and drug–drug interactions, and inconsistencies between study designs and medication dosages. Additional research is required to advance our understanding of the role of social cognition in schizophrenia, and to further establish the utility of targeted interventions in this setting.

## Introduction

The goal of treatment for schizophrenia is now “recovery,” rather than just the management of psychotic symptoms. Recovery is a multidimensional concept involving clinical, psychological, and social aspects that broadly encompass occupational, educational and social activities, and the attainment of meaningful interpersonal relationships and independent living [1]. Clinical recovery (or “remission”) refers to the reduction of “symptoms” (e.g., delusions). Psychological recovery refers to developing ways to understand and cope with psychiatric experiences and thereby regain a sense of control and structure in life. Social recovery encompasses involvement in social and vocational activities, the formation of fulfilling social relationships, and gaining access to a good living environment. Precise definitions of recovery vary, but most include “criteria for symptom stability or freedom from psychiatric hospitalization, plus criteria for normalization of social and work/school functioning over a prescribed period of time” [2]. A meta-analysis of 50 studies demonstrated that only 13.5% of patients with schizophrenia and related psychoses attained recovery, when recovery was defined as “improvements in both clinical and social domains, with evidence that improvements in at least one of these two domains had persisted for ≥2 years” [3].

The link between neurocognition and functional outcomes in schizophrenia is well-established [4–7]. More recently, however, social cognition has been recognized as an important driver of functional outcomes in individuals with schizophrenia [8–11]. For example, in a study conducted in outpatients with schizophrenia that assessed how neurocognition and social cognition impact on interpersonal skills, cognitive factors predicted approximately 15% of variance in social skill, whereas social cognitive abilities predicted an additional 26% of variance [12, 13].

Social cognition relates to the emotional and cognitive processes required to assimilate the cognitive and behavioral patterns of other people [10, 14]. Social cognition has been described as “the ability to construct representations of the relations between oneself and others, and to use those representations flexibly to guide social behaviors” [15, 16], and defined as “the mental operations that underlie social interactions, including perceiving, interpreting, and generating responses to the intentions, dispositions, and behaviors of others” [17]. There is general consensus that social cognition and non-social neurocognition are distinct from each other empirically and neurobiologically, although they are related [10, 17–19]. Moreover, social cognition has been proposed to mediate the link between neurocognition and social functioning in schizophrenia [11] and, as such, is a key driver of recovery. Importantly, antipsychotic therapy targeting remission of clinical symptoms has demonstrated a limited impact on social cognition [20, 21], and there has therefore been an increasing drive to develop therapeutic strategies to specifically improve social cognition in schizophrenia [16]. Such strategies have not only shown promise in improving social functioning, but may also improve psychotic symptoms [16].

The purpose of this article is to review current evidence relating to social cognition in schizophrenia and its clinical implications, including interventions designed to target the core domains of social cognition in order to improve functional outcomes and thereby increase the likelihood of recovery.

## Methodology of literature review

An initial literature search was conducted in PubMed using the search terms “schizophrenia” AND “cognition” AND “social functioning,” limited to Title/Abstract, over a time period of the past 10 years. This search identified a total of 231 titles, 19 of which were excluded from further assessment, since they were either not in the English language, did not specifically relate to schizophrenia, involved small case reports (<5 patients), or were a commentary on a previous publication. Abstracts of the remaining articles were then manually assessed for evidence relating to the core domains of social cognition in schizophrenia (emotion processing, theory of mind [ToM], attributional bias, and social perception), and relevant articles were identified for detailed review.

## Core domains of social cognition in schizophrenia

Social cognition is a multidimensional construct comprising several domains. A meeting sponsored by the National Institute of Mental Health in 2006 initially defined five domains of social cognition: emotion processing, ToM, attributional bias, social perception, and social knowledge [17]. Subsequently, a comprehensive survey of experts conducted during the early phase of the Social Cognition Psychometric Evaluation (SCOPE) Study (which was designed to reach a consensus on the core domains of social cognition in schizophrenia, and to assess the psychometric properties of existing measures of social cognition and their potential utility in clinical trials), identified emotion processing, ToM, attributional bias, and social perception as the four key domains of social cognition (Table 1) [22].

**Table 1:** Key domains of social cognition and associated measures.

**Domain**	**Definition**	**Measures**
Emotion processing	The perception and use of emotions	Bell Lysaker Emotion Recognition Task (BLERT) [23] Penn Emotion Recognition Test (ER-40) [24] Diagnostic Analysis of Nonverbal Accuracy 2 (DANVA2) [25] Facial Emotion Discrimination Test (FEDT) [26] Facial Emotion Identification Test (FEIT) [26] Vocal Emotion Identification Task (VEIT) [26]
Theory of Mind (ToM)	The ability to represent the mental states of others, including the inference of intentions, dispositions, and/or beliefs	Reading the Mind in the Eyes Test [27] The Awareness of Social Inferences Test (TASIT) [28] Hinting Task [29] Adult Faux Pas [30] Brüne Picture Sequencing Task [31] Happe's Stories [32] Silent Animations [33] Visual Perspective Taking Task [34] Sally-Anne Test [35] Smarties Task [36]
Attributional bias/style	The way in which individuals explain the causes, or make sense, of social events or interactions	Ambiguous Intentions and Hostility Questionnaire (AIHQ) [37] Internal, Personal and Situational Attributions Questionnaire (IPSAQ) [38] Attributional Style Questionnaire (ASQ) [39] Balanced Attributional Style Questionnaire (BASQ) [40] Real Events Attributional Style Questionnaire (REASQ) [41]
Social perception	The decoding and interpretation of social cues in others	Relationships Across Domains (RAD) [42] Profile of Non-verbal Sensitivity (PONS) [43] Half Profile of Non-verbal Sensitivity (Half PONS) [44] Interpersonal Perception Task (IPT-15) [45] Social Cue Recognition Test (SCRT) [46] Situational Features Recognition Test (SFRT) [46] Wechsler Adult Intelligence Scales-Revised (WAIS-R) [47]

*Adapted from Couture et al. [9] and Pinkham et al. [22]*.

**Figure 1:**
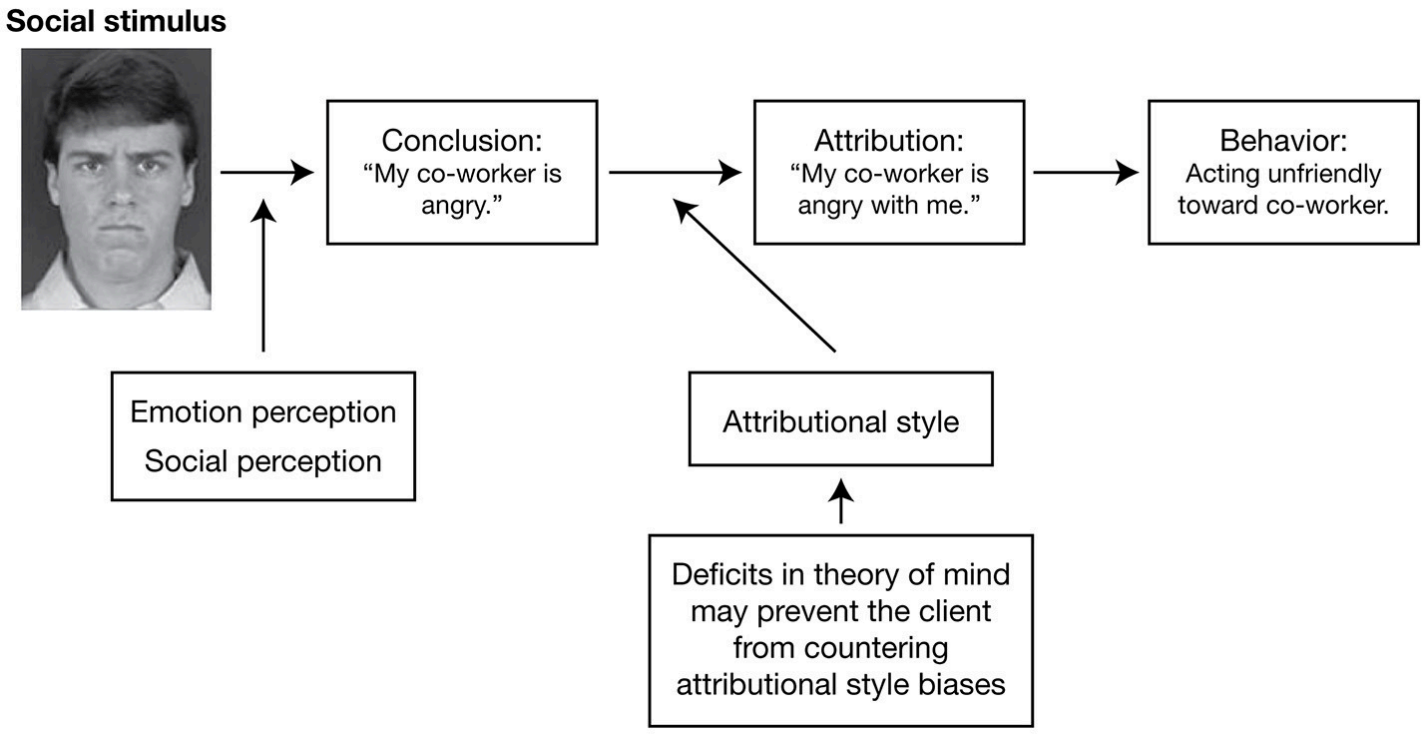
A conceptual model for understanding the interplay between the core domains of social cognition and social functioning. The model includes an example of a particular social situation (the reaction of someone with schizophrenia to a co-worker who has rushed past him without saying hello) in order to illustrate how this model would operate. Reproduced from Couture et al. [9] with permission from Oxford University Press.

Longitudinal studies have demonstrated that impairments in core domains of social cognition remain stable over time in patients with pre-first-episode, first-episode, early, and chronic phases of schizophrenia, and that these impairments are important determinants of functional outcome [48, 49]. As such, impairments in social cognition may represent important vulnerability indicators and targets for early clinical intervention [48, 49]. A conceptual model illustrating the four key domains of social cognition and their proposed links with functional outcome is presented in Figure 1 [9]. Evidence for the potential role of these domains in schizophrenia is outlined in the following sections.

### Emotion processing

Emotion processing (also known as emotion perception, emotion recognition, affect recognition, or affect perception) refers to the perception and use of emotional information [9, 17]. It includes three subdomains: emotion perception/recognition (i.e., the identification and recognition of emotion in others from facial expressions and/or non-face signals, such as voice [prosody]), understanding emotions, and managing emotions. Measures for emotion processing include the Penn Emotion Recognition Test (ER-40) [24] and Bell Lysaker Emotion Recognition Task (BLERT) [23] (Table 1). In the SCOPE Study, ER-40 and BLERT were among the measures showing the strongest psychometric properties and were therefore recommended for use in clinical trials [50].

Emotional prosody refers to the emotional quality of a person's voice, which is important for recognizing their emotional state and intention [51]. A meta-analysis of 17 studies conducted to evaluate the consistency and strength of emotional prosody impairment in schizophrenia found that the mean weighted effect size for the perception of emotional prosody was stable and significant (–1.24), and that this was also evident early on in the disease course [52]. The magnitude of this effect size suggests that impairments in emotional prosody perception may be particularly important among the cognitive deficits observed in schizophrenia [52]. The meta-analysis also found that *expression* of emotional prosody was significantly compromised in patients with schizophrenia (effect size, –1.11), although this finding was based on a smaller number of studies (*n* = 7) [52].

Emotion processing shows a consistent relationship with community functioning, which includes a wide range of activities and behaviors relating to social/work functioning and independent living [9, 53]. For example, in a study examining the role of emotion perception (assessed using the Facial Emotion Identification Test [FEIT]), neurocognition, psychiatric symptoms, and general visual perception as predictors of social mixing behaviors in inpatients with schizophrenia, emotion perception, and language abilities were found to correlate with adaptive social mixing behaviors, as judged by trained raters using the Social Behavior Scale [54].

A study conducted in individuals at ultra-high risk of psychosis, first-episode patients with schizophrenia, and healthy controls found that there were significant impairments in facial affect recognition (FAR) and vocal emotion recognition (assessed using a facial affect labeling test and an affective prosody recognition test, respectively) in both of the patient groups compared with the healthy controls, which remained significant regardless of sex, age, or educational background [55]. These results indicate that deficits in emotion recognition may precede the full expression of psychotic illness in schizophrenia [55]. Moreover, FAR deficits were identified in the asymptomatic offspring of parents with schizophrenia (i.e., “familial high-risk” individuals), when compared with age- and sex-matched healthy controls, and FAR accuracy scores were shown to predict parental rating of social skills in both groups [56]. Such findings indicate that identification of deficits in emotion processing in those at risk of schizophrenia could help inform early intervention [56].

FAR is among the most researched areas of social cognition in schizophrenia. Systematic reviews of studies investigating psychological interventions targeting FAR have demonstrated that such interventions are efficacious in improving FAR performance and functional status, but not psychotic symptoms [57, 58]. For example, a 6-week computerized intervention that included both cognitive training and FAR training was shown to significantly improve FAR and social functioning (assessed using the Personal and Social Performance scale [PSP]) in clinically stable male outpatients with schizophrenia [59]. However, there were no statistically significant differences between the intervention and control groups in changes in clinical symptoms (assessed using the Positive and Negative Syndrome Scale [PANSS]) or cognitive measures (assessed using the Hong Kong List Learning Test and the Letter-Number Sequencing Task) [59].

### ToM

ToM (also referred to as mentalizing, cognitive empathy, or mental state attribution) is defined as “the ability to represent the mental states of others, including the inference of intentions, dispositions, and/or beliefs” [60, 61]. Cognitive ToM relates to the capacity to interpret other people's beliefs; affective ToM, to the capacity to interpret other people's feelings [62]. Measures for ToM include Reading the Mind in the Eyes [27], The Awareness of Social Inferences Test (TASIT) [28], and the Hinting Task [29] (Table 1). The Hinting Task was one of the measures assessed in the SCOPE Study that showed the strongest psychometric properties and is therefore recommended for use in clinical trials [50].

Individuals with first-episode psychosis and chronic schizophrenia have been shown to have impaired ToM ability, compared with healthy controls and first-degree relatives [63–66]. Moreover, symptoms of schizophrenia have been shown to predict worse ToM ability [63], and impaired ToM ability has been shown to predict worse social and global functioning [63, 65], and to correlate with negative symptoms [65].

**Figure 2:**
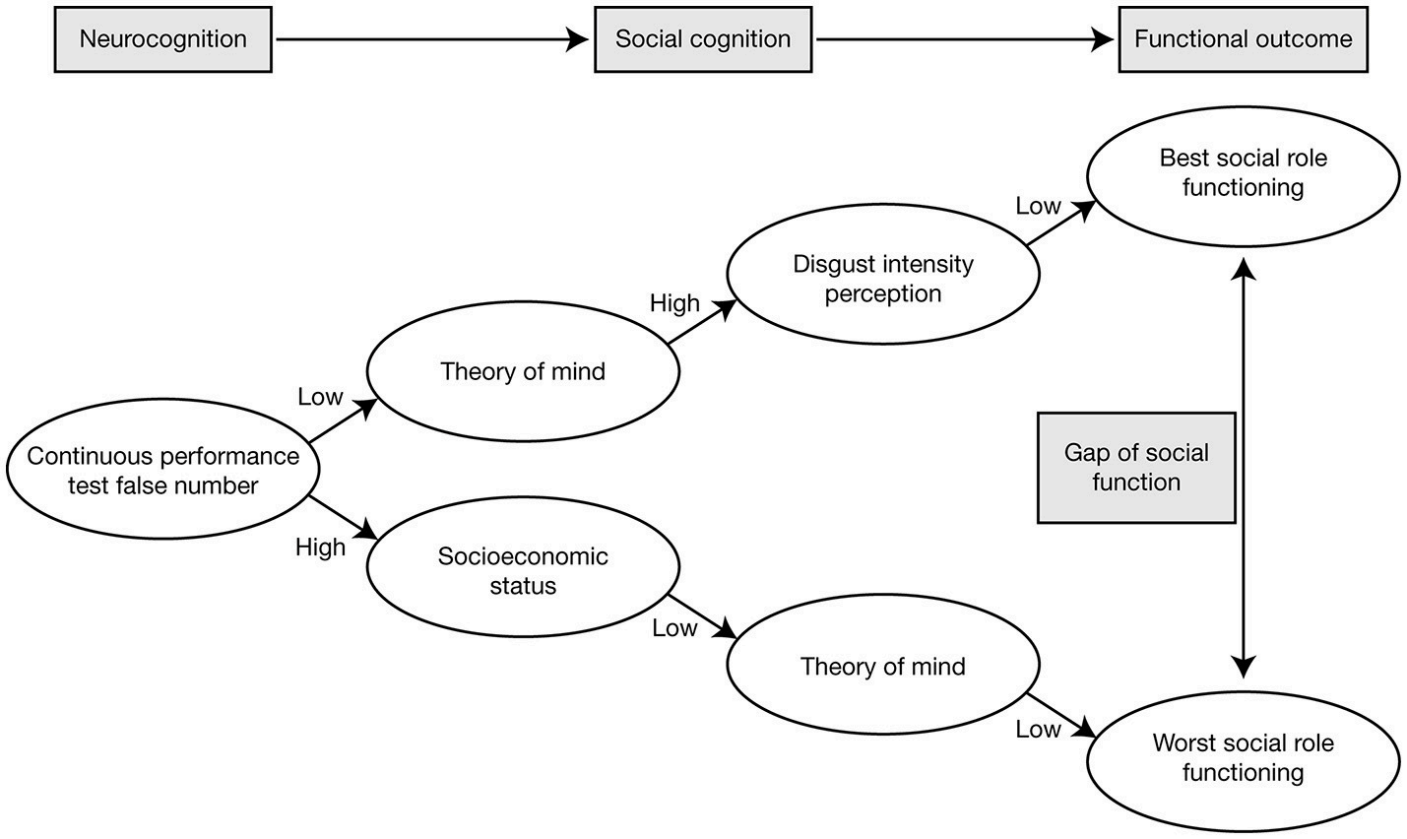
Pathways of the best and worst social role functioning in patients with schizophrenia, showing that social cognition mediates between neurocognition and functional outcomes. Reproduced from Bae et al. [70] under the terms of the CC BY non-commercial license.

In a study in which patients with schizophrenia were categorized as having low positive symptoms or moderate/severe positive symptoms, using a cut-off score of 14 on the PANSS positive subscale, there were no differences in ToM ability (assessed using Reading the Mind in the Eyes) between patients with low positive symptoms and healthy control subjects, but patients with moderate/severe positive symptoms performed significantly worse than patients with low positive symptoms and the healthy controls [67]. This could not be attributed simply to variation in patients' clinical state, since no such differences were observed when analogous categorizations were applied for PANSS negative symptoms and PANSS total score [67]. In a study designed to investigate the relationship between different domains of social cognition and psychotic symptomatology (assessed using PANSS) in a clinically stable population of outpatients with schizophrenia, ToM and mental state reasoning were found to be the strongest predictors of psychotic symptoms: mental state reasoning was best at predicting positive symptoms, the affective component of ToM was best at predicting negative symptoms, and cognitive ToM was best at predicting general psychotic symptoms [68]. Moreover, cognitive ToM (assessed using the Hinting Task) demonstrated strong correlations with multiple dimensions of social functioning (assessed using the Social Functioning Scale [SFS] [69]), including interpersonal communication, recreational activities, independence, and performance (Table 2) [68]. Another study that used data mining to explore predictors of social functioning in patients with schizophrenia found that good ToM, low sensitivity of disgust emotion (assessed as part of FAR), and good continuous attention were the factors resulting in the best social functioning [70]. Although, continuous attention was the strongest predictive factor, these findings provide evidence for social cognition as a mediator between neurocognition and functional outcomes, with the ability to significantly predict social functioning in patients with schizophrenia (Figure 2) [70].

**Table 2:** Pearson's bivariate correlations between measures of social cognition and social functioning in clinically stable outpatients with schizophrenia (*n* = 45).

**Measure**	**1**	**2**	**3**	**4**	**5**	**6**	**7**
1. FEDT	−						
2. FEIT	0.510^**^	−					
3. RMET	0.442^**^	0.534^**^	−				
4. Hinting Task	0.334^*^	0.207	0.346^*^	−			
5. UOT	0.003	0.041	0.365^*^	0.318^*^	−		
6. IPSAQ – EB	−0.026	−0.061	0.152	0.106	0.364^*^	−	
7. IPSAQ – PB	0.161	0.079	−0.049	0.223	−0.096	0.163	−
8. SFS – engagement	0.261	0.211	0.014	0.380^*^	0.206	0.000	0.228
9. SFS – interpersonal	0.169	−0.057	0.085	0.585^**^	0.227	0.083	0.051
10. SFS – prosocial	0.272	0.328^*^	0.312^*^	0.259	0.309^*^	0.047	0.059
11. SFS – recreation	0.197	0.081	0.188	0.401^**^	0.256	−0.003	−0.275
12. SFS – ind. & competence	0.168	0.093	0.290	0.280	0.203	0.188	0.104
13. SFS – ind. & performance	0.198	0.117	0.125	0.494^**^	0.247	−0.080	−0.070
14. SFS – employment	0.069	−0.002	0.074	0.170	0.085	−0.019	−0.177

*Significant correlations are shown in bold*.

* *Correlation is significant at the 0.05 level (two-tailed)*.

** *Correlation is significant at the 0.01 level (two-tailed)*.

EB, Externalizing Bias; FEDT, Facial Emotion Discrimination Test; FEIT, Facial Emotion Identification Test; IPFSQ, Internal, Personal, and Situational Attributions Questionnaire;

*PB, Personalizing Bias; RMET, Reading the Mind in the Eyes Test-revised; SFS, Social Functioning Scale; UOT, Unexpected Outcomes Test*.

*Reproduced from Brown et al. [68] with permission from Elsevier*.

An assessment of the associations between mental state attribution (i.e., ToM) and neurocognition, social skills, and clinical symptomatology in individuals with schizophrenia revealed that the best cognitive predictor of social skills was a combined verbal and non-verbal score of mental state attribution [71]. This effect was not mediated by neurocognition (i.e., executive planning skills). Mental state attribution was the only significant cognitive predictor of social skills, although levels of disorganized and negative symptoms were found to predict large proportions of variance in social functioning [71].

An investigation that used voxel-based morphometry and a battery of behavioral assessments of ToM processing indicated that ToM deficits in patients with schizophrenia may be related to a reduction in ventromedial prefrontal cortex gray matter volume [72]. However, it is currently not known whether loss of ventromedial prefrontal cortex gray matter could cause poor ToM skills, or whether the social isolation experienced by those with schizophrenia, and the consequent loss of opportunities to employ ToM skills, could cause loss of ventromedial prefrontal cortex gray matter over time [72]. This potentially has important therapeutic implications, since interventions aimed at improving ToM skills might, in theory, mitigate gray matter loss, and restore ventromedial prefrontal cortex function [72].

Individuals at ultra-high risk of schizophrenia have also been shown to have significant impairments in ToM abilities, compared with healthy controls [73]. Furthermore, ToM ability—but not emotion recognition, social perception, or attributional style—has been shown to be significantly correlated with current role and global functioning (assessed using the Global Functioning Social and Role scales, and the Social and Occupational Functioning Assessment Scale) in individuals at ultra-high risk of psychosis [74]. Evidence has also indicated that ToM deficits (confined to comprehension of higher-order false belief) emerge in subjects with at-risk mental state [75]. Adolescents who have a first-degree relative with schizophrenia (“genetic high risk” adolescents) have social impairments that increase the likelihood of later disease onset [76]. In a study that evaluated the psychometric properties of a theoretically-derived assessment of social functioning in genetic high risk adolescents (the “High-Risk Social Challenge” task), genetic high risk adolescents showed social skills impairments, compared with healthy controls, but did not display deficits in ToM [77]. ToM was assessed using the Reading the Mind in the Eyes Test-Revised Version (which measures the decoding component of ToM), and the authors speculate whether tools that assess the reasoning rather than the decoding aspect of ToM might better elucidate the role of ToM as a marker of vulnerability for later developing schizophrenia [77]. Although results from such studies are somewhat mixed, taken together they may indicate a role for specific preventative strategies targeting ToM at the prodromal stage.

ToM deficits also appear to affect patients' parenting ability. In a study investigating the association between functional ability in the parental role (i.e., active involvement and affective relationship) and cognitive performance, and level of insight and motivation, individuals with schizophrenia who were parents underwent comprehensive assessments for neurocognition, social cognition (ToM, emotion processing, social perception, and attributional bias), motivation and insight, and social functioning (using the Groningen Social Disabilities Schedule) [78]. The results demonstrated that deficits in first- and second-order ToM were significantly associated with parental role dysfunction, as were cognitive flexibility, speed of processing, and motivation [78]. Second-order ToM was found to be a specific predictor of parental role on logistic regression analysis [78].

Poor insight—defined as “the awareness of having a mental disorder, and of its symptoms and implications”—has been linked to poor outcome in schizophrenia [79]. In a study investigating the relationships between insight and ToM, an association was found between awareness of illness and ToM (specifically, the ability to understand the intentions of others on the Hinting Task), which was independent of other illness-related variables (neurocognition and clinical symptoms) [80]. Moreover, ToM was shown to be a mediator linking neurocognition with awareness of illness. In contrast, no association was observed between ToM and cognitive insight [80]. These findings indicate that social cognition interventions that target ToM skills, such as perspective-taking, could potentially improve awareness of illness and functional outcome in schizophrenia [80].

### Attributional bias/style

Attributional bias/style describes how individuals make sense/explain the causes of the positive and negative social events and interactions encountered in life, which is thought to have a significant impact on behaviors [13, 17, 61]. Key measures of attributional bias/style include the Ambiguous Intentions and Hostility Questionnaire (AIHQ) [37], Internal, Personal and Situational Attributions Questionnaire (IPSAQ) [38], and Attributional Style Questionnaire (ASQ) [39] (Table 1). IPSAQ measures a causal locus (external-personal vs. external-situational), which is not present in AIHQ and ASQ.

Some studies have specifically investigated the role of attributional bias in persecutory delusions in schizophrenia. A comparison of attributional bias (assessed using the IPSAQ) in patients with early psychosis and gender- and age-matched controls found that although a high proportion of the patients had persecutory delusions, they did not differ from the controls in terms of personalizing or externalizing bias [81]. In a cross-sectional study in which attributional bias was assessed using a revised version of the IPSAQ (IPAQ-R) in patients with persistent positive symptoms of schizophrenia and healthy controls, there were no differences between the healthy controls and either the overall group of patients with schizophrenia or the subgroup of patients with persecutory delusions, in terms of personalizing or externalizing biases [82]. Persecutory delusions were independently predicted by personalizing bias for negative events and depression, but these only accounted for 5% of the observed variance in persecutory delusions [82]. Patients with schizophrenia and the subgroup with persecutory delusions both displayed a self-blaming attributional style, tending to attribute negative events to themselves [82].

In a study comparing attributional style (assessed using the AIHQ), FAR (assessed using the FEIT, Facial Emotion Discrimination Test, and ER-40) and ToM (assessed using the Hinting Task) in outpatients with schizophrenia, outpatients with bipolar disorder, and a group of healthy controls, the clinical groups demonstrated hostile social cognitive biases, in comparison with the control group [64]. Patients with schizophrenia also showed poorer ToM ability than the patients with bipolar disorder and healthy controls [64]. Moreover, in patients with schizophrenia, a tendency to aggressiveness and PANSS score were the factors most strongly associated with global functioning [64]. Similarly, in a study into the factor structure of social cognition in schizophrenia, and the correlation between these factors and symptoms, neurocognition, and functional outcome, hostile attributional style was found to be significantly associated with PANSS positive and emotional discomfort factors, as well as PANSS total score [83]. This indicated that higher hostile attribution ratings (such as an increased tendency to report blame/hostility/aggression in response to ambiguous social situations) were correlated with higher levels of positive symptoms, anxiety, depression, and general emotional discomfort [83]. Hostile attributional style also approached significance in predicting quality of life (assessed using the Quality of Life Scale) [83]. Taken together, these findings indicate that hostile attributional style is an important driver of functional outcomes in schizophrenia and therefore a potential candidate for targeted intervention.

### Social perception

Social perception refers to “the decoding and interpretation of social cues in others” [22]. It includes the ability to integrate contextual information and social knowledge into judgment about other people's behaviors (e.g., being able to discern that two people are romantically involved, without knowing or interacting with them) [13]. Measures of social perception include Relationships Across Domains [42] (Table 1).

Social perception has been shown to be related to social behavior, social problem solving, and community functioning, and to mediate the relationship between neurocognition and functional outcome [9, 17, 84]. Poor social perception has also been associated with an inability to form trustingschizophrenia outpatients compared MSCT (relationships and improve quality of life [16, 85]. Social perception may therefore be a rational target for interventions aiming to enhance functional improvements [17].

In individuals with schizophrenia, social perception, and emotion responsivity were found to be positively correlated with functional outcome [86]. Emotion responsivity for positive and negative stimuli were shown to be slightly reduced in patients with schizophrenia, compared with controls, but the relationship between emotional responsivity, and functional outcome did not appear to be mediated by social perception [86]. These findings indicate that it may be important to take account of how a patient responds emotionally to supposed positive or rewarding events, in addition to their social cognitive abilities, when considering interventions aiming to improve functional outcomes [86].

## Interventions targeting social cognition in schizophrenia

### Social cognition training programs

Social cognition training programs aim to correct the specific social cognitive impairments associated with schizophrenia that are related to social functioning and readily transferable to real-world situations [16]. Such programs have additionally been found to indirectly result in improvements in clinical (positive and negative) symptoms, vocational prospects, and quality of life [16]. Broad-based interventions designed to improve functioning (such as Integrated Psychological Therapy [87], Integrated Neurocognitive Therapy [88], and Cognitive Enhancement Therapy [89]) have shown limited success in schizophrenia, but have helped inform the development of more targeted interventions [16], as outlined in the next section. In general, cognitive remediation programs are most effective in enhancing functional outcomes when integrated with psychosocial rehabilitation programs, by allowing individuals to practice cognitive skills in real-world settings [90].

#### Interventions targeting multiple social cognition domains

##### Social cognition and interaction training (SCIT)

SCIT is a 20-week, manualized, group intervention targeting emotion perception, ToM, and attributional bias [91]. It comprises three phases: emotion training, figuring out situations, and integration [91]. Pilot studies conducted in the inpatient setting reported improvements in ToM, attributions for ambiguous situations, and emotional and social perception following SCIT, as well as improvements in aggressive behaviors and self-reported social relationships [92, 93]. Effects on social functioning were sustained over 6 months in a follow-up study [94].

A modified version of SCIT (comprising twice-weekly sessions for 8 weeks) was compared with treatment as usual in an inpatient forensic ward in a randomized, single-blind, feasibility study [95]. The SCIT group demonstrated a significant improvement in FAR compared with the group that received treatment as usual [95]. In the outpatient setting, preliminary data from a quasi-experimental study comparing SCIT plus treatment as usual vs. treatment as usual alone indicated SCIT-related improvements in emotion perception [96]. A subsequent randomized controlled trial did not show significant improvement in emotional perception, but improvements in hostile attributional bias and social functioning were reported [97]. In the community setting, an initial study demonstrated the transportability, acceptability, and feasibility of SCIT, and there were indications of improvements in ToM and emotion perception (but not attributional bias) following SCIT [98]. Feasibility studies conducted in China and Finland have demonstrated that translated versions of SCIT are acceptable and effective in improving social cognition and social functioning [99, 100].

A 14-week pilot study investigated the impact of once-weekly family-assisted SCIT, compared with social stimulation once every 3 weeks, on quality of life, social functioning, and social cognition in clinically stable schizophrenia outpatients [101]. When pre-randomization assessments were compared with assessments after 16 weeks, patients who had received family-assisted SCIT demonstrated significant improvements in quality of life, social cognition, and social functioning; by contrast, results for nearly all outcome parameters declined in those who received social stimulation [101].

##### Metacognitive and social cognition training (MSCT)

MSCT was designed to “both remediate deficits and correct biases in social cognition” [102]. A preliminary efficacy study conducted in clinically stable schizophrenia outpatients compared MSCT (administered as 18 sessions over 10 weeks) with treatment as usual [102]. MSCT resulted in significant improvements in ToM, social perception, emotion recognition, and social functioning [102]. In addition, MSCT significantly reduced the tendency to jump to conclusions [102].

##### Social cognition training program (PECS in Spanish)

PECS was shown to improve some areas of ToM (measured using the Hinting Task), as well as the emotion recognition of sadness, anger, fear, and disgust, in outpatients with schizophrenia [103, 104].

#### Interventions targeting specific social cognition domains

##### Training of affect recognition (TAR)

TAR is a manualized, computer-aided, 12-session program primarily targeting deficits in FAR [105]. Patients with schizophrenia who have undergone TAR training have demonstrated improvements in FAR [106]. These patients were also shown to pay more attention to areas of the face that display emotion; however, this did not correlate with improvements in FAR performance [106].

A study comparing TAR training with Cognitive Remediation Training (CRT; primarily designed to target neurocognition) demonstrated that prosodic affect recognition, ToM, and social competence improved significantly more with TAR vs. CRT, and these improvements were reflected by a trend toward improvement in global social functioning [107]. By contrast, CRT only improved targeted neurocognitive areas, such as executive function, working memory, and attention [107].

##### GAÏA s-face (schizophrenia-facial affects recognition cognitive enhancement)

GAÏA s-face is an individual, computer-assisted cognitive remediation therapy designed to focus on FAR, which is administered as three 1-h sessions per week for 10 weeks [108, 109]. A single-blind study conducted in patients with stable schizophrenia compared GAÏA s-face with COgnitive REmediation in Schizophrenia (RECOS) [109]. RECOS is an individual neurocognitive remediation therapy targeting one to three out of six neurocognitive functions (verbal memory, working memory, executive functions, memory, and visuo–spatial attention, selective attention, and processing speed), according to each patient's cognitive and clinical profile [110]. RECOS also consists of three 1-h sessions per week and was administered for 10 weeks [109]. Both interventions resulted in a significant improvement in FAR performance, with a significantly greater effect observed with GAÏA s-face, compared with RECOS [109]. Clinical symptoms (assessed using PANSS) and social functioning (assessed using Echelle d'Autonomie Sociale, a social autonomy scale) also improved in the GAÏA s-face arm, but not in the RECOS arm [109].

##### Emotion and ToM imitation training (ETIT)

ETIT is an imitation treatment that was designed to improve social cognition and social functioning in patients with schizophrenia [111]. It comprises four phases: observing the gaze of people in photographs, imitating facial expressions, inferring an individual's mental state in a social situation, and attributing intentions by watching people's actions in a series of comic strips [111]. Preliminary data from a study conducted in outpatients with schizophrenia demonstrated that those who underwent ETIT improved on social cognitive measures, including emotion recognition and ToM, and showed better social functioning than those who underwent Problem Solving Training (the control group) [111]. The effects of rehabilitation training on neuro-physiological activation were assessed using the event-related potentials method, and an increase in electroactivity in the medio-frontal areas was only observed following ETIT, supporting the observed benefits on social cognition [111].

##### Emotion processing and ToM video-based training

A pilot study evaluated the practicality and effectiveness of a 12-week emotion processing and ToM video-based training program, compared with standard social cognitive rehabilitation treatment, in outpatients with schizophrenia [112]. Significant improvement in ToM abilities was demonstrated following video-based training, but there were no changes in emotion processing [112].

##### Mind reading: an interactive guide to emotions (MRIGE)

MRIGE is an interactive computerized program (comprising video clips, audio clips, and brief stories) that was originally developed to improve emotion and facial recognition in patients with autism spectrum disorders [113]. In a study conducted in patients with stable schizophrenia or schizoaffective disorder, the addition of MRIGE to a commercially available computerized cognitive remediation program (COGPACK) over 12 weeks was shown to significantly improve emotion processing (assessed using FEIT and FEDT), in comparison with cognitive remediation alone [114]. MRIGE plus cognitive remediation also resulted in significantly greater improvements in cognitive function (assessed using the MATRICS Consensus Cognitive Battery) and social functioning (assessed using PSP) than cognitive remediation alone [114].

##### ToM intervention (ToMI)

ToMI employs comic strips and faux pas stories to train cognitive and affective ToM [115]. A study conducted in outpatients with schizophrenia demonstrated improvement in ToM post-ToMI, compared with an active control group [115].

##### Visual and audio emotion processing training

An integrated multisensory approach, aiming to enhance emotion detection using either video or audio channels, was assessed in outpatients with schizophrenia and compared with an active control group [116]. Video training comprised short videos depicting human social interactions, selected from TASIT, with the audio and subtitles turned off. Audio training used only the audio component of the same videos. In the active control group, patients were involved in a newspaper discussion group. All three interventions were conducted in a 1-hourly session per week over 8 weeks [116]. Emotion recognition was assessed using FEIT (to evaluate visual recognition of emotion expression) and the Montreal Affective Voices test (to evaluate emotion expressed via audio) [116]. Significant improvements in both aspects of emotion processing were observed following training, and positive correlations were found between working memory (assessed using the Italian version of the Brief Assessment of Cognition in Schizophrenia), social functioning (assessed using PSP), and emotion processing [116].

##### “SoCog” mental-state reasoning training (SoCog-MSRT) and “SoCog” emotion recognition training (SoCog-ERT)

SoCog-MSRT is designed to target ToM and attribution style, but does not directly target emotion recognition [117]. SoCog-ERT combines the use of Ekman's Micro Expression Training Tool CD^1^ with activities and games designed to extend and strengthen recognition of the most important facial features [118]. SoCog-MSRT and SoCog-ERT were assessed in a pilot study conducted in patients with schizophrenia or schizoaffective disorder; both were administered as 12 bi-weekly sessions over 6 weeks [118]. SoCog-MSRT and SoCog-ERT both resulted in improvements in scores on a false belief reasoning task and the Reading the Mind in the Eyes test [118]. SoCog-ERT, but not SoCog-MSRT, improved emotion recognition, while SoCog-MSRT reduced biases in a small subgroup of patients with a personalizing bias [118].

### Pharmacological interventions for social cognition

#### Oxytocin (OXT)

OXT is a neuropeptide that interacts with a variety of neuromodulators, including serotonin and dopamine, in the nucleus accumbens, and amygdala, respectively [119]. In healthy controls, OXT has demonstrated beneficial effects on a range of social cognition domains and measures of social functioning [119].

In a cross-sectional study conducted in patients with schizophrenia and healthy controls, there were significant correlations between OXT plasma levels and social cognitive bias in the control group and in patients with delusions, but these were not observed in patients without delusions [120]. A significant correlation between social cognitive capacity and OXT plasma levels was only found in patients with delusions [120]. There is also some evidence to suggest that genetic variants of the OXT receptor may play a role in the social cognitive impairments observed in schizophrenia [121, 122].

The therapeutic use of intranasal OXT to improve social cognition and social functioning in schizophrenia has yielded mixed results [119, 123]. In a 6-week, placebo-controlled, double-blind pilot study, patients who received intranasal OXT experienced within-group improvements in perspective taking and the ability to recognize fear, and also an improvement in negative symptoms [124]. A small, randomized, placebo-controlled trial demonstrated that twice-daily intranasal OXT treatment for 14 days improved ToM and social perception in patients with schizophrenia [125]. In a small, randomized, within-subjects, placebo-controlled study designed to investigate whether a single dose of OXT could improve higher-order and lower-order social cognition, patients with schizophrenia received a single dose of oxytocin nasal spray (24 IU) and a placebo, administered 2 weeks apart [126]. OXT was shown to enhance performance on higher-order social cognition tasks (which assess social cognitive processing within the context of social communication), but had no effect on general neurocognition [126]. Improvement was greatest on tests that measured the appreciation of indirect hints and recognition of social faux pas [126]. In a 12-week, randomized, controlled trial, outpatients with schizophrenia or schizoaffective disorder received twice-daily intranasal OXT (24 IU) or placebo [127]. Their social cognitive function was assessed using the ER-40, Brüne Theory of Mind, Reading the Mind in the Eyes test, Trustworthiness task, and Ambiguous Intentions Hostility Questionnaire, measured at baseline, 6 weeks, and 12 weeks [127]. In addition, social function was assessed using the Specific Levels of Functioning Scale and a role-play test, and psychopathology was assessed using PANSS [127]. No evidence of beneficial effects on social cognition was observed for OXT compared with placebo [127]. OXT was slightly more beneficial than placebo on a component of social functioning, but there was also evidence that placebo was more beneficial than OXT on the role-play task [127]. In the schizophrenia subgroup, OXT resulted in a significant within-group reduction in PANSS negative symptoms and a significant between-group improvement in negative symptoms [127]. A randomized, double-blind, placebo-controlled trial investigated the efficacy of an extended treatment of OXT nasal spray combined with social cognition training to improve social cognition, clinical symptoms, and social functioning in young people with early psychosis [128]. Participants received OXT (24 IU) or placebo nasal spray twice daily for 6 weeks, combined with group social cognition training (two × 1-h sessions/week for 6 weeks), and an additional dose of OXT was administered before each weekly session [128]. Primary outcome measures were Reading the Mind in the Eyes Test, PANSS, and the SFS; assessments were conducted at baseline, post-treatment, and at 3-month follow-up [128]. No benefit of OXT nasal spray treatment vs. placebo was found [128]. In another randomized, double-blind, placebo-controlled trial, intranasal OXT did not modify jumping to conclusions in stable, medicated patients with schizophrenia [129].

The mixed findings observed in studies investigating the therapeutic potential of OXT in schizophrenia may be due to the impact of factors including task-specific effects, patient effects (e.g., age, sex, genetic variation in the OXT receptor, ancestry), and pharmacological effects (e.g., bioavailability, pharmacokinetics, neurotransmitter–drug interactions) [130].

#### Antipsychotics

Several studies have specifically explored the effects of antipsychotic treatment on social cognition and social functioning. An 8-week, randomized, multicenter, open-label study examined the effects of aripiprazole, and risperidone on social cognition and neurocognition in patients with schizophrenia [131]. Both treatments resulted in improvements in social cognitive and neurocognitive test scores, and reaction time [131]. The agents differed little on (social) cognitive test scores [131]. However, aripiprazole was significantly superior compared with risperidone on symbol substitution and reaction times for emotional working memory and working memory, and these improvements were shown to correlate with social functioning [131].

A 6-month, open-label, randomized, controlled pilot study compared the effects of risperidone long-acting injection and paliperidone palmitate on non-acute-phase social functioning in patients with schizophrenia [132]. Assessments at baseline and 6 months included the SFS (primary outcome), University of California San Diego Performance-Based Skills Assessment Brief (UPSA-B), Social Emotional Cognition Task (SECT), PANSS, and Drug-Induced Extrapyramidal Symptoms Scale (DIEPSS) [132]. Paliperidone palmitate was significantly more effective than risperidone long-acting injection on change from baseline in SFS total score, and the SFS competence and performance subscales scores [132]. The treatment groups did not differ significantly on change from baseline in UPSA-B, SECT, PANSS, and DIEPSS [132].

Patients participating in a 6-month, randomized, double-blind clinical trial comparing olanzapine, and quetiapine were assessed for improvements in social cognition and social functioning [133]. Social cognition was assessed using signal detection analysis of performance on the Social Cue Recognition Test [133]. In both treatment groups, there were modest, but significant, improvements on three out of four social cognition subscales [133].

Evidence regarding the relative impacts of antipsychotic treatments on social cognition in schizophrenia is currently inconclusive, due to inconsistencies in study designs, methodologies, and medication dosages [134]. Despite the lack of definitive evidence, it is rational to consider the wider impact of antipsychotics on cognition when selecting treatment [135, 136].

#### Raloxifene

Raloxifene is a first-generation selective estrogen receptor modulator that acts as an estrogen receptor agonist in the brain (and bone), and as an antagonist in other tissues [137]. Raloxifene has been shown to have a beneficial impact on attention/processing speed and memory in men and women with schizophrenia [138]. A 13-week, randomized, double-blind, placebo-controlled, crossover trial examined the effects of adjunctive raloxifene treatment (120 mg/day) on abnormal neural activity during angry FAR in schizophrenia [139]. Adjunctive raloxifene was found to significantly increase activation in the right hippocampus and left inferior frontal gyrus, compared with placebo, indicating that it may reverse abnormal neural activity during FAR, and suggesting a potential modifying role for estrogen in schizophrenia [139].

## Summary

Research in social cognition is gaining significant importance in schizophrenia. However, the complexity of the subject remains challenging. The past few decades have seen concerted multidisciplinary efforts from different fields, including neuroscience, psychiatry, psychology, computer sciences, anthropology, and philosophy, which have markedly changed the ways in which we conceptualize how knowledge is acquired, processed, and used. This area is also highly relevant to clinical practice, since impairments in social cognition are consistently found in patients with schizophrenia. There is increasing evidence that social cognition is a direct predictor of functional outcomes, particularly community functioning. Similarly, the concepts of neurocognition and social cognition are interlinked, with social cognition mediating the relationship between basic neurocognition and functional outcome, thereby making it central to daily life functioning. Several psychosocial interventions have shown promise in overcoming and correcting impairments in social cognition associated with schizophrenia. Indeed, current evidence indicates that most of the targeted social cognitive training programs that have been developed to date may produce improvements in the domains of social cognition for which they are designed. Further research is required to advance our understanding of the role of social cognition in schizophrenia, and to further establish the utility of targeted interventions in this setting.

## Author contributions

AJ and AC made substantial contributions to the conception of this article, and the analysis and interpretation of data it contains; were involved in drafting the article or revising it critically for important intellectual content; provided final approval of the version to be published; and agreed to be accountable for all aspects of the work in ensuring that questions related to the accuracy or integrity of any part of the work were appropriately investigated and resolved.

### Conflict of interest statement

AJ has received speaker fees from, and undertaken consultancy work and the organization of scientific meetings for, Sunovion and Lundbeck, over the past 3 years. AC declares that the research was conducted in the absence of any commercial or financial relationships that could be construed as a potential conflict of interest.

## Footnotes

^1^http://www.paulekman.com/micro-expressions/

## Abbreviations

AIHQ: Ambiguous Intentions and Hostility Questionnaire
BASQ: Balanced Attributional Style Questionnaire
BLERT: Bell Lysaker Emotion Recognition Task
CRT: Cognitive Remediation Training
DIEPSS: Drug-Induced Extrapyramidal Symptoms Scale
ER-40: Penn Emotion Recognition Test
ETIT: Emotion and ToM Imitation Training
FAR: facial affect recognition
FEIT: Facial Emotion Identification Test
GAÏA s-face: Schizophrenia-Facial Affects recognition Cognitive Enhancement
MRIGE: Mind Reading: An Interactive Guide to Emotions
MSCT: Metacognitive and Social Cognition Training
OXT: oxytocin
PANSS: Positive And Negative Syndrome Scale
PSP: Personal and Social Performance scale
REASQ: Real Events Attributional Style Questionnaire
RECOS: COgnitive REmediation in Schizophrenia
SCIT: Social Cognition and Interaction Training
SCOPE: Social Cognition Psychometric Evaluation study
SECT: Social Emotional Cognition Task
SFS: Social Functioning Scale
SoCog-ERT: ‘SoCog’ Emotion Recognition Training
SoCog-MSRT: ‘SoCog’ Mental-State Reasoning Training
TAR: Training of Affect Recognition
TASIT: The Awareness of Social Inferences Test
ToM: Theory of Mind
ToMI: Theory of Mind Intervention
UPSA-B: University of California San Diego Performance-Based Skills Assessment Brief.
